# Characterizing Early Changes in Quality of Life in Young Women With Breast Cancer

**DOI:** 10.3389/fpsyg.2022.871194

**Published:** 2022-05-06

**Authors:** Hend M. Al-Kaylani, Bradley T. Loeffler, Sarah L. Mott, Melissa Curry, Sneha Phadke, Ellen van der Plas

**Affiliations:** ^1^Department of Psychiatry, University of Iowa Hospital and Clinics, Iowa City, IA, United States; ^2^Holden Comprehensive Cancer Center, University of Iowa Hospital and Clinics, Iowa City, IA, United States; ^3^Division of Hematology, Oncology, and Blood and Marrow Transplantation, Department of Internal Medicine, Carver College of Medicine, University of Iowa, Iowa City, IA, United States

**Keywords:** health related quality of life, survivorship, young adult, breast cancer, risk factors

## Abstract

**Introduction:**

Younger age at diagnosis is a risk factor for poor health-related quality of life (HRQOL) in long-term breast cancer survivors. However, few studies have specifically addressed HRQOL in young adults with breast cancer (i.e., diagnosed prior to age 40), nor have early changes in HRQOL been fully characterized.

**Methods:**

Eligible female patients with breast cancer were identified through our local cancer center. To establish HRQOL, patients completed the Functional Assessment of Cancer Therapy-Breast (FACT-B) around diagnosis and 12 months later. Sociodemographic factors, genetic susceptibility to cancer, tumor- and treatment-related factors, and comorbidities (e.g., depression/anxiety) were abstracted from medical records and the local oncology registry. Mixed-effects models were used to identify changes in FACT-B scores during the first year of treatment and to determine whether any demographic/treatment-related factors modulated changes in scores.

**Results:**

Health-related quality of life in young patients with breast cancer was within normal limits at baseline, with a FACT-B overall well-being score of 108.5 (95% confidence limits [CI] = 103.7, 113.3). Participants reported slight improvements over a 12-month period: FACT-B overall well-being scores increased 6.6 points (95% CI = 2.1, 11.1, *p* < 0.01), functional well-being improved 3.0 points (95% CI = 2.0, 4.1, *p* < 0.01), emotional well-being improved 1.9 points (95% CI = 0.9, 2.8, *p* < 0.01), and physical well-being improved 1.5 points (95% CI = 0.2, 2.8, *p* = 0.03), on average. Participants with anxiety/depression at baseline reported greater improvements in FACT-B overall well-being (change: 12.9, 95% CI = 6.4, 9.5) and functional well-being (change: 5.2, 95% CI = 3.5, 6.9) than participants who did not have anxiety/depression at baseline (change in FACT-B overall well-being: 4.9, 95% CI = 0.2, 9.7; change in functional well-being: 2.3, 95% CI = 1.1, 3.4). Marital status, reconstructive surgery, and baseline clinical staging were also significantly associated with changes in aspects of HRQOL, although their impact on change was relatively minimal.

**Conclusion:**

Young women with breast cancer do not report HRQOL concerns during the first year of treatment. Improvements in HRQOL during the first year of treatment may be attributable to a sense of relief that the cancer is being treated, which, in the short run, may outweigh the negative late effects of treatment.

## Introduction

Approximately 5% of new cancer diagnoses in the United States occur in adolescents and young adults (AYA) who are between 15 and 39 years old (SEER, 2022) at the time of diagnosis. Breast cancer accounts for up to 30% of cancer diagnoses among young women (Miller et al., 2020; Scott et al., 2020; Cathcart-Rake et al., 2021) and often presents aggressively with a higher frequency of adverse histopathological characteristics, worse prognosis, and higher risk of recurrence (Gewefel and Salhia, 2014; Johnson et al., 2018). In 2020, the 5-year relative survival rate for young women breast cancer was estimated to be 86% (Miller et al., 2020), making survivorship an important consideration. Research in AYA cancer survivorship has intensified recently (Smith et al., 2013; Nichols et al., 2021), although a considerable amount of what is published about AYA cancer survivorship has been extrapolated from childhood cancer survivor cohorts (Prasad et al., 2015; Jacola et al., 2016; Miller et al., 2020). As a result, survivorship research specific to young women with breast cancer is lagging.

Health-related quality of life (HRQOL), a broad concept encompassing the perceived physical and mental health of individuals (CDC, 2021), is an important endpoint in clinical trials (Haslam et al., 2020). Women with breast cancer reported the highest prevalence of unmet needs in survivorship (Miroševič et al., 2019), underscoring the urgency of continued research on HRQOL in this population. Younger age at breast cancer diagnosis has been identified as a risk factor for reduced HRQOL in breast cancer survivors (Ganz et al., 2003). For instance, Champion et al. (2014) showed that women diagnosed prior to the age of 45 years were more likely to report depressive symptoms, fatigue, and more attention problems than women diagnosed after age 45. Another study reported that female breast cancer survivors who were diagnosed between 35 and 50 years old had a steeper decline in HRQOL in the first 3 years after treatment and recovered more slowly compared with survivors who were diagnosed at an older age (Roine et al., 2021). Notably, while younger age at diagnosis has been established as a risk factor for reduced HRQOL in breast cancer survivors, HRQOL in young adult breast cancer was not specifically addressed. Patients with breast cancer who are in their 20 and 30 s may experience different social and physical challenges than older patients (Quinn et al., 2015), highlighting the importance of studying HRQOL in young women with breast cancer.

A variety of factors besides younger age have been implicated in poor long-term HRQOL in breast cancer survivors. These factors range from somatic predictors (e.g., fatigue, upper extremity lymphedema, obesity, and menopausal symptoms such as hot flashes and sleep disturbances) (Rossi and Pagani, 2017; Saha et al., 2017; Schmidt et al., 2019; Dominici et al., 2021; Jørgensen et al., 2021; Lei et al., 2021; Park et al., 2021) to social factors (e.g., lower socioeconomic status, lack of private insurance, and race/ethnicity) (Ashing-Giwa and Lim, 2009; Derouen et al., 2013; Samuel et al., 2016; Claridy et al., 2018; Dominici et al., 2021) and mental health concerns (Van Esch et al., 2012; Schoormans et al., 2015; Carreira et al., 2018). While HRQOL in breast cancer survivors of all ages may be impacted by these factors, some predictors are more pertinent to younger survivors. For instance, concerns about premature menopause and infertility are more common among survivors diagnosed prior to age 51 and adversely affect HRQOL (Howard-Anderson et al., 2012). Relatedly, young patients with breast cancer typically require intensive therapy due to aggressive tumors that are often diagnosed at a later, more advanced stage (Murphy et al., 2019; Cathcart-Rake et al., 2021). The associated side effects of intense treatment regimens pose a greater risk of poor physical and emotional well-being (Samuel et al., 2016). Dominici et al. (2021) showed that long-term HRQOL was dependent on local therapy strategy in breast cancer survivors who were diagnosed prior to age 40. Young women who underwent more extensive surgeries (e.g., unilateral/bilateral mastectomies) reported lower HRQOL than peers who received breast-conserving therapy.

Finally, most research efforts to date have focused on long-term outcomes (Champion et al., 2014; Cohee et al., 2017; Dominici et al., 2021; Roine et al., 2021). As a result, little is known about HRQOL in the early stages of survivorship. Research conducted in the first year of treatment across a variety of cancers in AYAs identified an unmet need for mental health or fertility counseling services (Quinn et al., 2015), disruptions in work (Schmidt et al., 2019), educational pursuits (Parsons et al., 2012), and parenting (Bailey et al., 2010). The needs of patients and survivors change as they adjust to their cancer diagnosis and survivorship (Costanzo et al., 2007; Husson et al., 2017). Characterizing patterns of HRQOL and factors associated with reduced HRQOL in AYA breast cancer could improve preventative care and patient empowerment (Howard-Anderson et al., 2012; Gewefel and Salhia, 2014).

In summary, there is a knowledge gap regarding early changes in HRQOL in young women with breast cancer, limiting effective surveillance of HRQOL in this population. To address this gap, we aimed to characterize HRQOL in the first year following diagnosis in women with breast cancer who were diagnosed between 18 and 39 years old. We also sought to explore the impact of patient- and treatment-related factors on modulating HRQOL. Given findings of poor HRQOL in long-term breast cancer survivors (Champion et al., 2014; Roine et al., 2021), we hypothesized that HRQOL would decline during the first year of treatment.

## Materials and Methods

### Participants

**FIGURE 1:**
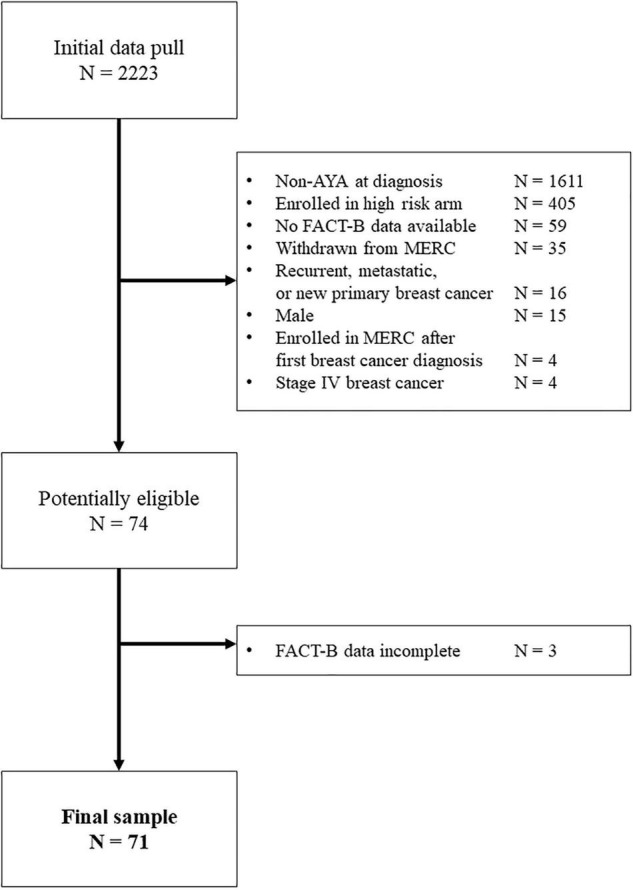
Consort diagram.

Participants were identified through the Breast Molecular Epidemiological Resource Core (BMER) data repository at the Holden Comprehensive Cancer Center, University of Iowa Hospitals and Clinics. Patients were eligible to enroll in BMER once they received a definitive breast cancer diagnosis (e.g., primary cancer, recurrence, or metastatic disease) and up until 1 year post-diagnosis. For the current study, patients enrolled in BMER who were female and were diagnosed between ages 18 and 39 were eligible. Those who experienced metastatic and relapsed cancers were excluded, however, of 74 potentially eligible participants, 71 (96%) were included in the final sample (Figure 1).

### Standard Protocol Approvals, Registrations, and Patient Consents

Participants gave written informed consent prior to enrolling in the BMER study. Directly identifiable information was removed from the data. This project was approved by the Institutional Review Board for Human Subjects Research at the University of Iowa (ID 202106560).

### Outcome Measure

Health-related quality of life was ascertained with the Functional Assessment of Cancer Therapy—Breast (FACT-B). This measure is composed of the Functional Assessment of Cancer Therapy—General (FACT-G) (Cella et al., 1993) and 10 additional questions that are specific to a breast cancer subscale score (BCS). The FACT-B is widely used in breast cancer-specific samples and shows high reliability (Brady et al., 1997).

The Functional Assessment of Cancer Therapy—General provides an overall well-being score, as well as scores for physical well-being (PWB), social well-being (SWB), emotional well-being (EWB), and functional well-being (FWB). The FACT-G overall well-being scores range from 0 to 108, with 108 indicating excellent well-being. Since the FACT-B includes the breast cancer-specific questions of the BCS, the maximum value for FACT-B overall well-being is 148. Questionnaires were mailed or emailed to patients prior to their clinic appointment. Responses were recorded on a five-point Likert scale, and higher scores represent better quality of life.

### Predictors

#### Patient Characteristics

Patient variables of interest included (1) sociodemographic factors (i.e., age at diagnosis; insurance status at diagnosis [private/public]; race/ethnicity; and marital status [married/not married]); (2) family history of cancers [no/yes]; (3) tumor- and treatment-related factors (i.e., estrogen receptor status [positive/negative]; progesterone receptor status [positive/negative]; HER2 status [positive/negative]; and fertility counseling [no/yes]); and (4) comorbidities (i.e., body mass index [BMI] and depression/anxiety at the time of cancer diagnosis [no/yes]). Note that insurance status was used as a proxy for socioeconomic status. Further, anxiety/depression was determined by retrieving ICD codes for the presence of anxiety/depression or by identifying whether the individual had an active prescription of antidepressants or anxiolytics at the time of diagnosis.

#### Medical Parameters

Relevant medical variables were abstracted from medical records and the oncology registry at the University of Iowa Hospitals and Clinics and included the following: (1) clinical staging [0–I/II–III]; (2) laterality [left/right]; (3) surgery type [lumpectomy/mastectomy]; (4) reconstruction surgery [no/yes]; (5) chemotherapy [no/yes]; (6) targeted therapy [no/yes]; (7) radiation [no/yes]; (8) hormone therapy [no/yes]; and (9) ovarian suppression [no/yes].

### Statistical Approach

For descriptive purposes, mean baseline scores of participants were plotted with published normative means obtained from the FACT-G in US adults (*n* = 1,075) and adults with cancer (*n* = 2,236; Brucker et al., 2005).

Changes in FACT-B subscale scores were estimated using linear mixed-effects models. Random effects were included to account for the longitudinally correlated nature of repeated HRQOL assessments at unequal time spacing between visits with a spatial power correlation structure.

Linear mixed-effects models were also used to measure the association between the rate of change in FACT-B subscale scores and baseline clinical, comorbidity, and treatment characteristics. Random effects were similarly included to account for repeated measures with unequal spacing between visits. All statistical testing was two-sided and assessed for significance at the 5% level using SAS v9.4 (SAS Institute, Cary, NC, United States). Plots were generated in R.

## Results

### Sample

A total of 71 women had completed a FACT-B questionnaire at enrollment (mean 1.5 months from diagnosis) and/or a questionnaire 12 months after enrollment (mean 11.4 months) in which at least one subscale was complete. The median age at diagnosis was 35.0 years (range 22.0–39.0). Table 1 presents the demographic and treatment information.

**TABLE 1:** Sample characteristics.

Variable	Levels	Sample *N* = 71
**Patient characteristics**		
*Socio-demographic factors*		
Age at diagnosis		**Median (range)**
		35.0 (22.0–39.0)
		**Frequency (%)**
Race	American Indian or Alaska Native	1 (1.4)
	Asian	3 (4.2)
	Other	3 (4.2)
	White	64 (90.1)
Ethnicity	Hispanic	2 (2.8)
	Non-Hispanic	69 (97.2)
Insurance status	Medicaid	4 (5.7)
	None	4 (5.7)
	Private	62 (88.6)
	Missing	1
Marital status	Married	49 (74.2)
	Not Married	17 (25.8)
	Missing	5
*Genetic susceptibility*		
Family history of cancer	No	31 (43.7)
	Yes	40 (56.3)
*Hormone receptor status*		
Estrogen receptor status	Negative	33 (46.5)
	Positive	38 (53.5)
Progesterone receptor status	Negative	36 (51.4)
	Positive	34 (48.6)
	Missing	1
HER2 status	Negative	44 (68.8)
	Positive	20 (31.3)
	Missing	7
Fertility counseling	No	51 (71.8)
	Yes	20 (28.2)
*Comorbidities*		
Anxiety/depression at diagnosis	No	50 (71.4)
	Yes	20 (28.6)
	Missing	1
BMI		Median (range)
		25.1 (18.4–41.8)
** Medical parameters**		
Baseline clinical stage	0–II	22 (33.8)
	II–III	43 (66.2)
	Missing	6
Laterality of tumor	Left	39 (54.9)
	Right	32 (45.1)
Surgery type	Lumpectomy	17 (23.9)
	Mastectomy	54 (76.1)
Reconstruction surgery	No	29 (40.8)
	Yes	42 (59.2)
Chemotherapy	No	13 (18.3)
	Yes	58 (81.7)
Targeted therapy	No	51 (71.8)
	Yes	20 (28.2)
Radiation	No	33 (46.5)
	Yes	38 (53.5)
Hormone therapy	No	42 (59.2)
	Yes	29 (40.8)
Ovarian suppression	No	60 (84.5)
	Yes	11 (15.5)

### Health-Related Quality of Life

**FIGURE 2:**
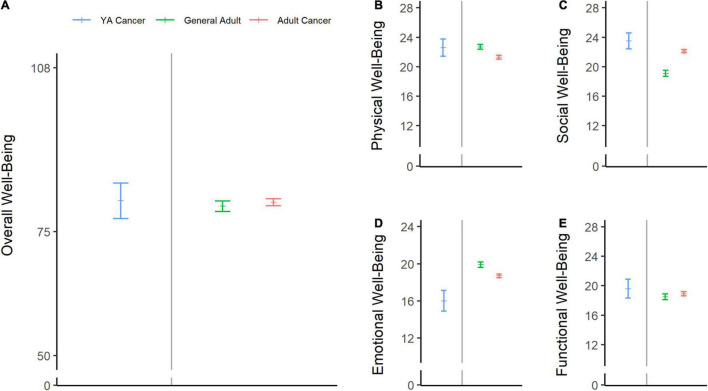
Baseline health-related quality of life (HRQOL) scores in adolescents and young adult (AYA) patients with breast cancer compared with normative data. The sample’s mean scores and 95% confidence limits of the means are shown in blue for overall well-being **(A)**, physical well-being **(B)**, social well-being **(C)**, emotional well-being **(D)**, and functional well-being **(E)**. Green lines represent the means of the general US adult population (ages 18–91), and pink lines represent the scores from a sample of adult patients with cancer (ages 18–92) (Brucker et al., 2005).

Descriptive statistics for FACT-B subscale scores at baseline and at 12 months post-diagnosis are shown in Table 2. HRQOL at baseline was within normal limits relative to a normative sample of adults and adults with cancer in the United States (Brucker et al., 2005; Figure 2A–E), although young patients with breast cancer reported lower emotional well-being than the reference samples (Figure 2D).

**TABLE 2:** FACT-B scores at baseline and at 12 months post-diagnosis.

Covariate	Baseline	Change	*P*-value
		(12 months–Enrollment)	
	Mean (95% CI)	Mean (95% CI)	
Overall well-being	108.5 (103.7, 113.3)	6.6 (2.1, 11.1)	<0.01
Physical well-being	22.6 (21.4, 23.8)	1.5 (0.2, 2.8)	0.03
Social well-being	23.5 (22.4, 24.6)	−0.8 (−1.9, 0.3)	0.13
Emotional well-being	16.0 (14.9, 17.1)	1.9 (0.9, 2.8)	<0.01
Functional well-being	19.6 (18.3, 20.9)	3.0 (2.0, 4.1)	<0.01
Breast Cancer Subscale	27.4 (25.7, 29.1)	−0.1 (−2.1, 1.8)	0.88

**FIGURE 3:**
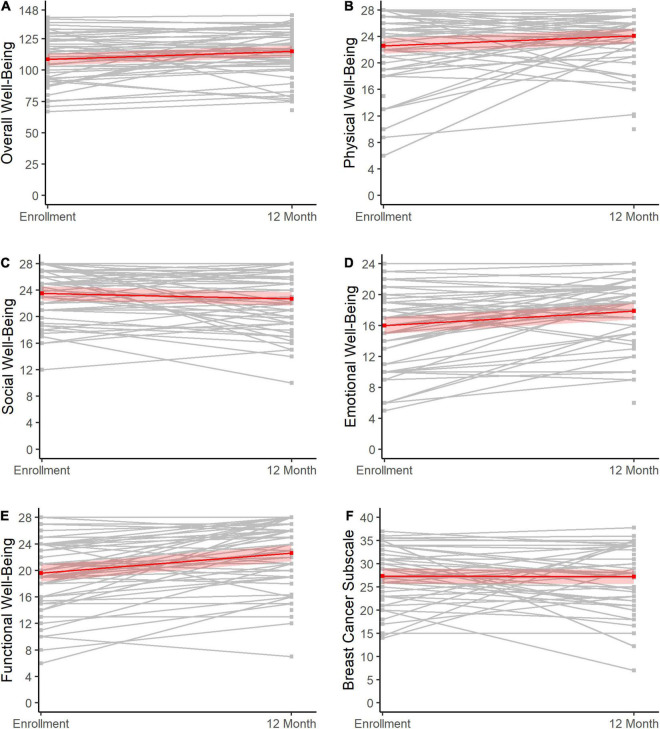
Change in HRQOL from baseline to 12 months post-diagnosis. Changes in HRQOL from enrollment to 12 months post-diagnosis (X-axes) are shown for overall well-being **(A)**, physical well-being **(B)**, social well-being **(C)**, emotional well-being **(D)**, functional well-being **(E)**, and scores on the breast cancer subscale **(F)**. The gray lines represent the individuals, while the red lines show the overall trends across the sample with 95% confidence limits.

Across scales, there was minimal change in scores from baseline to the 12-month assessment. Generally, a slight increase in scores was evident (Table 2 and Figures 3A–F), which reached statistical significance for FACT-B overall well-being (change score: 6.6, 95% CI = 2.1, 11.1, *p* < 0.01; Figure 3A), functional well-being (change score: 3.0, 95% CI = 2.0, 4.1, *p* < 0.01; Figure 3B), emotional well-being (change score: 1.9, 95% CI = 0.9, 2.8, *p* < 0.01; Figure 3C), and physical well-being (change score: 1.5, 95% CI = 0.2, 2.8, *p* = 0.03; Figure 3E).

### Predictors of Change in Health-Related Quality of Life

Estimated means and rates of change for each predictor across scales are shown in Supplementary Tables 1–6.

**FIGURE 4:**
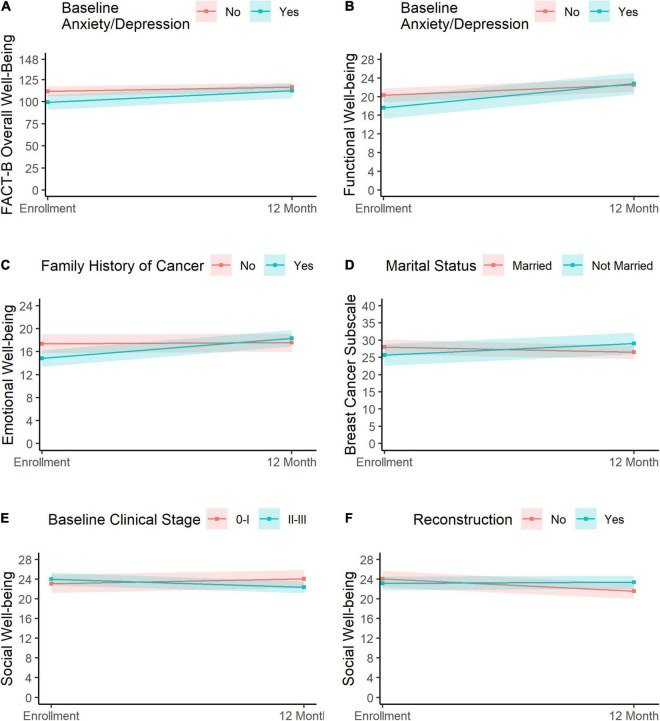
Predictors of change in HRQOL. Change in overall well-being and functional well-being varied significantly as a function of anxiety/depression (**A,B**, respectively), where the anxious/depressed group (blue) showed greater improvement than the non-anxious/depressed group (pink). Change in emotional well-being is shown in panel **(C)**. Participants with a family history of cancer (blue) showed a greater improvement than those without a history of cancer (pink). Panel **(D)** depicts changes in breast cancer scores for married patients (pink) and non-married patients (blue), with the latter showing slight improvements relative to the former. Changes in social well-being varied as a function of baseline clinical stage **(E)** and reconstruction **(F)**. Participants with stage II–III cancer (**E**, blue) exhibited a reduction in social well-being relative to participants with stage 0–I clinical stage (**E**, pink). Finally, participants who did not undergo reconstruction surgery (**F**, pink) reported a slight decline in social well-being relative to participants who did have reconstruction surgery (**F**, blue).

Anxiety/depression at diagnosis was significantly associated with the rate of change in FACT-B overall well-being, where the anxious/depressed group showed a greater increase in FACT-B overall well-being (change: 12.9, 95% CI = 6.4, 19.5) than the group that was not anxious/depressed (change: 4.9, 95% CI = 0.2, 9.7; Figure 4A). A similar trend for anxiety/depression status was observed for functional well-being, with the anxious/depressed group exhibiting a greater increase (change: 5.2, 95% CI = 3.5, 6.9) than the non-anxious/depressed group (change: 2.3, 95% CI = 1.1, 3.4; Figure 4B).

Changes in emotional well-being varied significantly as a function of family history of cancer, where those with a family history of cancer exhibited greater improvements in emotional well-being (change: 3.5, 95% CI = 2.2, 4.7) than participants who did not have a family history of cancer (change: 0.2, 95% CI = −1.1, 1.4; Figure 4C).

Change in the breast cancer subscale scores varied significantly as a function of marital status, where patients who were married exhibited a slight decrease (change: −1.5, 95% CI = −4.1, 1.0), while those who were not married at baseline reported some improvement (3.3, 95% CI = 0.0, 6.7, Figure 4D).

Clinical staging (0–I vs. II–III) and reconstruction (no vs. yes) were both associated with change in social well-being. First, patients with stage II–III cancer exhibited a slight decline in social well-being scores (change: −1.7, 95% CI = −3.0, −0.3), while those with stage 0–I cancer showed a slight improvement (change: 1.0, 95% CI = −1.1, 3.1; Figure 4E). Likewise, patients who did not undergo reconstruction surgery showed a slight decrease in social well-being (change: −2.5, 95% CI = −4.1, −0.9), while those who had undergone reconstructive surgery reported relatively stable social well-being over the assessment period (change: 0.2, 95% CI = −1.1, 1.5; Figure 4F). Results were consistent upon inclusion of both clinical staging and reconstruction in a multivariable model.

## Discussion

Young women who were diagnosed with breast cancer in young adulthood report excellent HRQOL at diagnosis and even exhibit some improvement over a 12-month period. Our results suggest deficits in HRQOL may not emerge until later in survivorship. There is strong evidence for impaired long-term HRQOL in breast cancer survivors, particularly those who were diagnosed at a younger age (Kroenke et al., 2004; Howard-Anderson et al., 2012; Champion et al., 2014; Carreira et al., 2018; Maurer et al., 2021; Park et al., 2021). The results from the present study are in line with recent work in early breast cancer (Criscitiello et al., 2021), although others have reported declines in HRQOL during treatment in general cohorts of patients with breast cancer (Schoormans et al., 2015; Maurer et al., 2021). Improvements in HRQOL during treatment have also been reported in pediatric cancer populations who undergo intense treatment regimens (Mitchell et al., 2016; Garas et al., 2019). As with long-term breast cancer survivors, pediatric cancer survivors also exhibit poor HRQOL later in survivorship (Kunin-Batson et al., 2014; van der Plas et al., 2020). It seems reasonable to expect that initial improvements in HRQOL are attributable to a sense of relief that the cancer is being treated (Garas et al., 2019), which, in the short run, may outweigh the negative late effects of treatment (Criscitiello et al., 2021). Our results suggest that young adult breast cancer survivors may share this relief to some extent during the first year of treatment and survivorship. However, it is possible that HRQOL diminishes further into survivorship due, in part, to changes in perceived support and increased cumulative burden of treatment-related late effects.

Perceived support from family, friends, and healthcare professionals is an important factor in HRQOL in cancer patients (Usta, 2012; Hurtado-de-Mendoza et al., 2021) and appears to modulate the perceived severity of chemotherapy-related symptoms (Oh et al., 2020). There is evidence to suggest that perceived social support may diminish the further patients that are from diagnosis. A study by Arora et al. (2007) showed that patients with breast cancer reported high levels of emotional support from family and friends at diagnosis; however, emotional support had significantly declined at follow-up. It is possible that diminishing support contributes to decreased HRQOL further in survivorship of breast cancer survivors. Moreover, young breast cancer survivors (diagnosed < 50 years) with a small social network appear to be particularly vulnerable to low HRQOL in comparison with older survivors (Bloom et al., 2012).

Treatment for breast cancer has been associated with long-term neurocognitive difficulties that are often referred to as “chemo-brain” (Janelsins et al., 2017; Ketterl et al., 2019; Eide and Feng, 2020; Schroyen et al., 2021). Neurocognitive difficulties may encompass memory loss, difficulty concentrating, and other relatively subtle changes that can have a detrimental impact on daily functioning (Chan et al., 2016; Sousa et al., 2020) and are negatively associated with HRQOL (Kunin-Batson et al., 2014; Eide and Feng, 2020; van der Plas et al., 2020; Wagner et al., 2020). AYA cancer survivors across various types of cancers have identified neurocognitive deficits as a key issue (Jim et al., 2018). It is possible that functional difficulties of cancer treatment do not manifest until later in survivorship, even though physiological brain changes are already afoot. In their systematic review, Sousa et al. (2020) reported that there was no evidence for functional change acutely after chemotherapy for breast cancer, even though brain changes were already noted. Others showed that alterations in blood markers of inflammation and neuronal integrity increased the patients with longer breast cancer that are from diagnosis (Schroyen et al., 2021). The notion of “allostatic load” may explain the lag between physiological changes and functional deficits. Allostatic load conveys the cumulative impact of physiological “wear and tear” on the brain and body that eventually results in deterioration in physical and mental health (Suvarna et al., 2020; Schroyen et al., 2021). Thus, a longitudinal assessment of a wide variety of markers indicative of physiological “wear and tear” (e.g., quantitative neuroimaging, blood markers of brain injury, and subtle cognitive decline) is a promising tool to help preserve HRQOL in young breast cancer survivors.

The present study also explored factors that may modulate HRQOL in young women with breast cancer, including sociodemographic factors, genetic susceptibility, hormone receptor status, comorbidities, and treatment-related factors. Anxiety/depression was significantly associated with a change in overall HRQOL and functional well-being. Critically, our target population intersects at two demographics that report an elevated risk of anxiety and depression in cancer survivorship: young age and female sex (Yi and Syrjala, 2017). Approximately 28% of patients had anxiety/depression at diagnosis, which could conceivably have contributed to lower emotional well-being at baseline in this sample (Figure 2D). Somewhat surprisingly, patients with anxiety/depression reported overall improvement in HRQOL relative to patients who did not have anxiety/depression at baseline. Young women with anxiety/depression reported lower emotional well-being at baseline and essentially caught up with the non-anxious/non-depressed group 1 year later. One potential explanation is patients with anxiety/depression may have difficulty with emotion regulation in stressful situations (Jazaieri et al., 2015), resulting in greater mood fluctuations than in non-anxious/non-depressed patients. Furthermore, it is possible that the observed improvements in anxiety/depression are related to factors that were not assessed here, such as physical exercise (Patsou et al., 2018). As described above, these improvements may be temporary. Van Esch et al. (2012) reported that anxiety symptoms at breast cancer diagnosis significantly predicted HRQOL 2 years after surgery. Another study showed that the negative impact of depression on HRQOL in breast cancer survivors increased with increased time from diagnosis (Schoormans et al., 2015). Continued surveillance is essential to ensure that these potentially vulnerable, young breast cancer survivors receive the appropriate care (Stafford et al., 2015; Carreira et al., 2018).

Other factors that modulated change in aspects of HRQOL included a family history of cancer, clinical staging at diagnosis, reconstruction, and marital status. Although significant associations were identified, none of these factors appeared to have a clinically meaningful impact on HRQOL during the first year of treatment. For instance, individuals with a family history of cancer reported an average increase of less than four points in emotional well-being. Trends were similar for the other factors that modulated change in HRQOL: Social well-being scores of young women with stage II–III cancer declined with less than two points from baseline to 12 months post-diagnosis, while that of the 0–I group increased one point. Notably, in their research on sociodemographic factors related to HRQOL in breast cancer survivors, Patsou et al. (2018) reported that marital status was not associated with depression/anxiety in survivors age 50 and younger; however, marital status did have a negative impact on depressive symptoms in older breast cancer survivors. These results highlight the possibility that the impact of potential risk factors varies based on age at diagnosis. Collectively, our findings suggest that patient and demographic factors have a minimal impact on changes in HRQOL early in the course of treatment and survivorship in young women with breast cancer.

### Limitations

The longitudinal nature of the analyses is a strength of the study, although several limitations warrant mentioning. First, our sample was limited in size, racial diversity, and socioeconomic diversity. These demographic limitations may restrict the generalizability of our findings and underscore the importance of independent replication of the results. Young, African-American breast cancer survivors reported a higher rate of unmet needs, financial distress, and lower physical/functional well-being relative to White breast cancer survivors (Samuel et al., 2016). Yet, African-American patients with breast cancer and survivors are vastly underrepresented in research (Samuel et al., 2016; Nolan et al., 2018), including our own. With a larger and more diverse sample, we can obtain a more holistic and intersectional evaluation of the risk factors that modulate young adult cancer survivorship. Prioritizing research in underrepresented groups will help reduce disparities in HRQOL (Hurtado-de-Mendoza et al., 2021).

Second, breast cancer is exceedingly uncommon among adolescents (SEER, 2022), and our results do not speak directly to adolescent cancer. The AYA age bracket spans a period of dynamic biological and psychosocial changes (Crone and van Duijvenvoorde, 2021). For instance, white matter volume increases rapidly in adolescence, but steady growth of regional white matter is still observed well into the third decade of life (Pomponio et al., 2020). Age-dependent differences in responsibilities, identities, and cognitive abilities also exist in this age bracket, likely resulting in varied survivorship experiences. Increased emphasis on patients’ neurodevelopmental stage may contribute to a better understanding of risk factors for reduced HRQOL.

Third, while the FACT-G is established as sensitive to change (Brady et al., 1997), a review by Luckett et al. (2011) notes that research on potential ceiling effects in the FACT-G is lacking. Constructing HRQOL measures with increased sensitivity to change while maintaining brevity for use in clinics can improve clinicians’ understanding of patient well-being, screen for patients at risk of decline, and identify promising interventions (Perry et al., 2007).

Relatedly, our results are based on patient-reported outcomes. While these types of assessments remain critical in understanding patient experiences, patient-reported measures have known limitations (McKenna, 2016). One of such limitations is response bias, which can encompass under- or over-reporting of problems (Burchett and Ben-Porath, 2019). As described earlier, broadening the scope and depth of assessments to encompass neuroimaging and blood-based biomarkers may further enhance our ability to appropriately address HRQOL.

## Conclusion

Health-related quality of life for AYA breast cancer survivors aligns with population norms and remains mostly stable during the first year of treatment. Patient- and treatment-related factors had a limited impact on change in HRQOL during the first year of treatment. While some significant associations were demonstrated (e.g., greater improvement in HRQOL among anxious/depressed patients relative to non-anxious/depressed patients), the clinical significance of these changes remains to be determined. Given that breast cancer at a young age has been clearly associated with poor long-term HRQOL, further study on this population is required to ensure adequate HRQOL is maintained in the long term.

## Data Availability Statement

The original contributions presented in the study are included in the article/Supplementary Material, further inquiries can be directed to the corresponding author.

## Ethics Statement

Written, informed consent was obtained from participants before enrollment in the BMER study. Directly identifiable information was removed from the data. This project was approved by the Institutional Review Board for Human Subjects Research at the University of Iowa (ID 202106560).

## Author Contributions

EP, HA, and SP contributed to the conceptualization, design, and methodology of the study. EP, HA, MC, and SM contributed to the investigation. BL and SM were performed statistical analysis. BL and EP visualized the data. HA and EP wrote the initial draft of the manuscript. SP and EP contributed to funding acquisition. All authors had full access to the data in the study, take responsibility for the integrity of the data and accuracy of the data analysis, revised, read, and approved the submitted version of the manuscript.

## Conflict of Interest

The authors declare that the research was conducted in the absence of any commercial or financial relationships that could be construed as a potential conflict of interest.

## Publisher’s Note

All claims expressed in this article are solely those of the authors and do not necessarily represent those of their affiliated organizations, or those of the publisher, the editors and the reviewers. Any product that may be evaluated in this article, or claim that may be made by its manufacturer, is not guaranteed or endorsed by the publisher.

## References

[B1] Arora N. K. Finney Rutten L. J. Gustafson D. H. Moser R. Hawkins R. P. (2007). Perceived helpfulness and impact of social support provided by family, friends, and health care providers to women newly diagnosed with breast cancer. *Psychooncology* 16 474–486. 10.1002/pon.1084 16986172

[B2] Ashing-Giwa K. T. Lim J. W. (2009). Examining the impact of socioeconomic status and socioecologic stress on physical and mental health quality of life among breast cancer survivors. *Oncol. Nurs. Forum* 36 79–88. 10.1188/09.onf.79-88 19136341

[B3] Bailey E. H. Pérez M. Aft R. L. Liu Y. Schootman M. Jeffe D. B. (2010). Impact of multiple caregiving roles on elevated depressed mood in early-stage breast cancer patients and same-age controls. *Breast Cancer Res. Treat.* 121 709–718. 10.1007/s10549-009-0645-1 19936914 PMC2869396

[B4] Bloom J. R. Stewart S. L. Oakley-Girvan I. Banks P. J. Shema S. (2012). Quality of life of younger breast cancer survivors: persistence of problems and sense of well-being. *Psychooncology* 21 655–665. 10.1002/pon.1965 21538677

[B5] Brady M. J. Cella D. F. Mo F. Bonomi A. E. Tulsky D. S. Lloyd S. R. (1997). Reliability and validity of the functional assessment of cancer therapy-breast quality-of-life instrument. *J. Clin. Oncol.* 15 974–986. 10.1200/jco.1997.15.3.974 9060536

[B6] Brucker P. S. Yost K. Cashy J. Webster K. Cella D. (2005). General population and cancer patient norms for the functional assessment of cancer therapy-general (FACT-G). *Eval. Health Prof.* 28 192–211. 10.1177/0163278705275341 15851773

[B7] Burchett D. Ben-Porath Y. S. (2019). Methodological considerations for developing and evaluating response bias indicators. *Psychol. Assess.* 31 1497–1511. 10.1037/pas0000680 31763874

[B8] Carreira H. Williams R. Müller M. Harewood R. Stanway S. Bhaskaran K. (2018). Associations between breast cancer survivorship and adverse mental health outcomes: a systematic review. *J. Natl. Cancer Inst.* 110 1311–1327. 10.1093/jnci/djy177 30403799 PMC6292797

[B9] Cathcart-Rake E. J. Ruddy K. J. Bleyer A. Johnson R. H. (2021). Breast cancer in adolescent and young adult women under the age of 40 years. *JCO Oncol. Pract.* 17:O2000793. 10.1200/op.20.00793 33449828

[B10] CDC (2021). *Health-Related Quality of Life (HRQOL).* Atlanta, GA: CDC.

[B11] Cella D. F. Tulsky D. S. Gray G. Sarafian B. Linn E. Bonomi A. (1993). The functional assessment of cancer therapy scale: development and validation of the general measure. *J. Clin. Oncol.* 11 570–579. 10.1200/jco.1993.11.3.570 8445433

[B12] Champion V. L. Wagner L. I. Monahan P. O. Daggy J. Smith L. Cohee A. (2014). Comparison of younger and older breast cancer survivors and age-matched controls on specific and overall quality of life domains. *Cancer* 120 2237–2246. 10.1002/cncr.28737 24891116 PMC4158315

[B13] Chan A. Ng T. Chan R. J. Poon E. Farid M. (2016). Are adolescent and young adult cancer patients affected by ‘chemobrain’?: a call for evidence. *Expert Rev. Qual. Life Cancer Care* 1 187–188. 10.1080/23809000.2016.1181977

[B14] Claridy M. D. Ansa B. Damus F. Alema-Mensah E. Smith S. A. (2018). Health-related quality of life of African-American female breast cancer survivors, survivors of other cancers, and those without cancer. *Qual. Life Res.* 27 2067–2075. 10.1007/s11136-018-1862-z 29704078 PMC6658173

[B15] Cohee A. A. Adams R. N. Johns S. A. Von Ah D. Zoppi K. Fife B. (2017). Long-term fear of recurrence in young breast cancer survivors and partners. *Psychooncology* 26 22–28. 10.1002/pon.4008 26490953 PMC4840075

[B16] Costanzo E. S. Lutgendorf S. K. Mattes M. L. Trehan S. Robinson C. B. Tewfik F. (2007). Adjusting to life after treatment: distress and quality of life following treatment for breast cancer. *Br. J. Cancer* 97 1625–1631. 10.1038/sj.bjc.6604091 18000503 PMC2360272

[B17] Criscitiello C. Spurden D. Piercy J. Rider A. Williams R. Mitra D. (2021). Health-related quality of life among patients with HR+/HER2- early breast cancer. *Clin. Ther.* 43 1228–1244.e4. 10.1016/j.clinthera.2021.04.020 34256965

[B18] Crone E. A. van Duijvenvoorde A. C. K. (2021). Multiple pathways of risk taking in adolescence. *Dev. Rev.* 62:100996. 10.1016/j.dr.2021.100996

[B19] Derouen M. C. Gomez S. L. Press D. J. Tao L. Kurian A. W. Keegan T. H. (2013). A population-based observational study of first-course treatment and survival for adolescent and young adult females with breast cancer. *J. Adolesc. Young Adult Oncol.* 2 95–103. 10.1089/jayao.2013.0004 24066271 PMC3779013

[B20] Dominici L. Hu J. Zheng Y. Kim H. J. King T. A. Ruddy K. J. (2021). Association of local therapy with quality-of-life outcomes in young women with breast cancer. *JAMA Surg.* 156:e213758. 10.1001/jamasurg.2021.3758 34468718 PMC8411359

[B21] Eide S. Feng Z. P. (2020). Doxorubicin chemotherapy-induced “chemo-brain”: meta-analysis. *Eur. J. Pharmacol.* 881 173078. 10.1016/j.ejphar.2020.173078 32505665

[B22] Ganz P. A. Greendale G. A. Petersen L. Kahn B. Bower J. E. (2003). Breast cancer in younger women: reproductive and late health effects of treatment. *J. Clin. Oncol.* 21 4184–4193. 10.1200/jco.2003.04.196 14615446

[B23] Garas A. McLean L. A. De Luca C. R. Downie P. McCarthy M. C. (2019). Health-related quality of life in paediatric patients up to five years post-treatment completion for acute lymphoblastic leukaemia: a systematic review. *Support Care Cancer* 27 4341–4351. 10.1007/s00520-019-04747-8 30900055

[B24] Gewefel H. Salhia B. (2014). Breast cancer in adolescent and young adult women. *Clin. Breast Cancer* 14 390–395. 10.1016/j.clbc.2014.06.002 25034440

[B25] Haslam A. Herrera-Perez D. Gill J. Prasad V. (2020). Patient experience captured by quality-of-life measurement in oncology clinical trials. *JAMA Netw. Open* 3:e200363. 10.1001/jamanetworkopen.2020.0363 32129865 PMC7057133

[B26] Howard-Anderson J. Ganz P. A. Bower J. E. Stanton A. L. (2012). Quality of life, fertility concerns, and behavioral health outcomes in younger breast cancer survivors: a systematic review. *J. Natl. Cancer Inst.* 104 386–405. 10.1093/jnci/djr541 22271773

[B27] Hurtado-de-Mendoza A. Gonzales F. Song M. Holmes E. J. Graves K. D. Retnam R. (2021). Association between aspects of social support and health-related quality of life domains among African American and White breast cancer survivors. *J. Cancer Surviv*. 10.1007/s11764-021-01119-2 [Epub ahead of print]. 34655040 PMC10166003

[B28] Husson O. Zebrack B. J. Block R. Embry L. Aguilar C. Hayes-Lattin B. (2017). Health-related quality of life in adolescent and young adult patients with cancer: a longitudinal study. *J. Clin. Oncol.* 35 652–659. 10.1200/jco.2016.69.7946 28095151

[B29] Jacola L. M. Edelstein K. Liu W. Pui C. H. Hayashi R. Kadan-Lottick N. S. (2016). Cognitive, behaviour, and academic functioning in adolescent and young adult survivors of childhood acute lymphoblastic leukaemia: a report from the childhood cancer survivor study. *Lancet Psychiatry* 3 965–972. 10.1016/S2215-0366(16)30283-8 27639661 PMC5056029

[B30] Janelsins M. C. Heckler C. E. Peppone L. J. Kamen C. Mustian K. M. Mohile S. G. (2017). Cognitive complaints in survivors of breast cancer after chemotherapy compared with age-matched controls: an analysis from a nationwide, multicenter, prospective longitudinal study. *J. Clin. Oncol.* 35 506–514. 10.1200/jco.2016.68.5826 28029304 PMC5455314

[B31] Jazaieri H. Morrison A. S. Goldin P. R. Gross J. J. (2015). The role of emotion and emotion regulation in social anxiety disorder. *Curr. Psychiatry Rep.* 17:531. 10.1007/s11920-014-0531-3 25413637

[B32] Jim H. S. L. Jennewein S. L. Quinn G. P. Reed D. R. Small B. J. (2018). Cognition in adolescent and young adults diagnosed with cancer: an understudied problem. *J. Clin. Oncol.* 36 2752–2754. 10.1200/jco.2018.78.0627 30040524 PMC7010417

[B33] Johnson R. H. Anders C. K. Litton J. K. Ruddy K. J. Bleyer A. (2018). Breast cancer in adolescents and young adults. *Pediatr. Blood Cancer* 65:e27397. 10.1002/pbc.27397 30156052 PMC6192832

[B34] Jørgensen M. G. Toyserkani N. M. Hansen F. G. Bygum A. Sørensen J. A. (2021). The impact of lymphedema on health-related quality of life up to 10 years after breast cancer treatment. *NPJ Breast Cancer* 7:70. 10.1038/s41523-021-00276-y 34075045 PMC8169644

[B35] Ketterl T. G. Syrjala K. L. Casillas J. Jacobs L. A. Palmer S. C. McCabe M. S. (2019). Lasting effects of cancer and its treatment on employment and finances in adolescent and young adult cancer survivors. *Cancer* 125 1908–1917. 10.1002/cncr.31985 30707763 PMC6508988

[B36] Kroenke C. H. Rosner B. Chen W. Y. Kawachi I. Colditz G. A. Holmes M. D. (2004). Functional impact of breast cancer by age at diagnosis. *J. Clin. Oncol.* 22 1849–1856. 10.1200/jco.2004.04.173 15143077

[B37] Kunin-Batson A. Kadan-Lottick N. Neglia J. P. (2014). The contribution of neurocognitive functioning to quality of life after childhood acute lymphoblastic leukemia. *Psychooncology* 23 692–699. 10.1002/pon.3470 24497266

[B38] Lei Y. Ho S. C. Kwok C. Cheng A. Cheung K. L. Lee R. (2021). Menopausal symptoms inversely associated with quality of life: findings from a 5-year longitudinal cohort in Chinese breast cancer survivors. *Menopause* 28 928–934. 10.1097/gme.0000000000001784 33878090

[B39] Luckett T. King M. T. Butow P. N. Oguchi M. Rankin N. Price M. A. (2011). Choosing between the EORTC QLQ-C30 and FACT-G for measuring health-related quality of life in cancer clinical research: issues, evidence and recommendations. *Ann. Oncol.* 22 2179–2190. 10.1093/annonc/mdq721 21339384

[B40] Maurer T. Thöne K. Obi N. Jung A. Y. Behrens S. Becher H. (2021). Health-related quality of life in a cohort of breast cancer survivors over more than 10 years post-diagnosis and in comparison to a control cohort. *Cancers (Basel)* 13:1854. 10.3390/cancers13081854 33924513 PMC8069882

[B41] McKenna S. P. (2016). The limitations of patient-reported outcome measurement in oncology. *J. Clin. Pathways* 2 37–46.

[B42] Miller K. D. Fidler-Benaoudia M. Keegan T. H. Hipp H. S. Jemal A. Siegel R. L. (2020). Cancer statistics for adolescents and young adults, 2020. *CA Cancer J. Clin.* 70 443–459. 10.3322/caac.21637 32940362

[B43] Miroševič Š Prins J. B. Selič P. Zaletel Kragelj L. Klemenc Ketiš Z. (2019). Prevalence and factors associated with unmet needs in post-treatment cancer survivors: a systematic review. *Eur J Cancer Care (Engl.)* 28 e13060. 10.1111/ecc.13060 31008544

[B44] Mitchell H. R. Lu X. Myers R. M. Sung L. Balsamo L. M. Carroll W. L. (2016). Prospective, longitudinal assessment of quality of life in children from diagnosis to 3 months off treatment for standard risk acute lymphoblastic leukemia: results of children’s oncology group study AALL0331. *Int. J. Cancer* 138 332–339. 10.1002/ijc.29708 26235006 PMC5138856

[B45] Murphy B. L. Day C. N. Hoskin T. L. Habermann E. B. Boughey J. C. (2019). Adolescents and young adults with breast cancer have more aggressive disease and treatment than patients in their forties. *Ann. Surg. Oncol.* 26 3920–3930. 10.1245/s10434-019-07653-9 31376035

[B46] Nichols H. B. Baggett C. D. Engel S. M. Getahun D. Anderson C. Cannizzaro N. T. (2021). The adolescent and young adult (AYA) horizon study: an AYA cancer survivorship Cohort. *Cancer Epidemiol. Biomarkers Prev.* 30 857–866. 10.1158/1055-9965.Epi-20-1315 33619021 PMC8102328

[B47] Nolan T. S. Frank J. Gisiger-Camata S. Meneses K. (2018). an integrative review of psychosocial concerns among young African American breast cancer survivors. *Cancer Nurs.* 41 139–155. 10.1097/ncc.0000000000000477 28221214

[B48] Oh G. H. Yeom C. W. Shim E. J. Jung D. Lee K. M. Son K. L. (2020). The effect of perceived social support on chemotherapy-related symptoms in patients with breast cancer: a prospective observational study. *J. Psychosom. Res.* 130:109911. 10.1016/j.jpsychores.2019.109911 31923732

[B49] Park J. Rodriguez J. L. O’Brien K. M. Nichols H. B. Hodgson M. E. Weinberg C. R. (2021). Health-related quality of life outcomes among breast cancer survivors. *Cancer* 127 1114–1125. 10.1002/cncr.33348 33237602 PMC8035208

[B50] Parsons H. M. Harlan L. C. Lynch C. F. Hamilton A. S. Wu X. C. Kato I. (2012). Impact of cancer on work and education among adolescent and young adult cancer survivors. *J. Clin. Oncol.* 30 2393–2400. 10.1200/jco.2011.39.6333 22614977 PMC3675694

[B51] Patsou E. D. Alexias G. T. Anagnostopoulos F. G. Karamouzis M. V. (2018). Physical activity and sociodemographic variables related to global health, quality of life, and psychological factors in breast cancer survivors. *Psychol. Res. Behav. Manag.* 11 371–381. 10.2147/prbm.S170027 30233264 PMC6134954

[B52] Perry S. Kowalski T. L. Chang C. H. (2007). Quality of life assessment in women with breast cancer: benefits, acceptability and utilization. *Health Qual. Life Outcomes* 5:24. 10.1186/1477-7525-5-24 17474993 PMC1877797

[B53] Pomponio R. Erus G. Habes M. Doshi J. Srinivasan D. Mamourian E. (2020). Harmonization of large MRI datasets for the analysis of brain imaging patterns throughout the lifespan. *Neuroimage* 208 116450. 10.1016/j.neuroimage.2019.116450 31821869 PMC6980790

[B54] Prasad P. K. Hardy K. K. Zhang N. Edelstein K. Srivastava D. Zeltzer L. (2015). Psychosocial and neurocognitive outcomes in adult survivors of adolescent and early young adult cancer: a report from the childhood cancer survivor study. *J. Clin. Oncol.* 33 2545–2552. 10.1200/jco.2014.57.7528 26150441 PMC4525049

[B55] Quinn G. P. Gonçalves V. Sehovic I. Bowman M. L. Reed D. R. (2015). Quality of life in adolescent and young adult cancer patients: a systematic review of the literature. *Patient Relat. Outcome Meas.* 6 19–51. 10.2147/prom.s51658 25733941 PMC4337625

[B56] Roine E. Sintonen H. Kellokumpu-Lehtinen P. L. Penttinen H. Utriainen M. Vehmanen L. (2021). Long-term health-related quality of life of breast cancer survivors remains impaired compared to the age-matched general population especially in young women. Results from the prospective controlled BREX exercise study. *Breast* 59 110–116. 10.1016/j.breast.2021.06.012 34225091 PMC8264211

[B57] Rossi L. Pagani O. (2017). Adjuvant endocrine therapy in breast cancer: evolving paradigms in premenopausal women. *Curr. Treat. Options Oncol.* 18:28. 10.1007/s11864-017-0473-1 28439796

[B58] Saha P. Regan M. M. Pagani O. Francis P. A. Walley B. A. Ribi K. (2017). Treatment efficacy, adherence, and quality of life among women younger than 35 years in the international breast cancer study group TEXT and SOFT adjuvant endocrine therapy trials. *J. Clin. Oncol.* 35 3113–3122. 10.1200/jco.2016.72.0946 28654365 PMC5597253

[B59] Samuel C. A. Pinheiro L. C. Reeder-Hayes K. E. Walker J. S. Corbie-Smith G. Fashaw S. A. (2016). To be young, Black, and living with breast cancer: a systematic review of health-related quality of life in young Black breast cancer survivors. *Breast Cancer Res. Treat.* 160 1–15. 10.1007/s10549-016-3963-0 27601138 PMC5111400

[B60] Schmidt M. E. Scherer S. Wiskemann J. Steindorf K. (2019). Return to work after breast cancer: the role of treatment-related side effects and potential impact on quality of life. *Eur. J. Cancer Care (Engl.)* 28:e13051. 10.1111/ecc.13051 31033073

[B61] Schoormans D. Czene K. Hall P. Brandberg Y. (2015). The impact of co-morbidity on health-related quality of life in breast cancer survivors and controls. *Acta Oncol.* 54 727–734. 10.3109/0284186x.2014.998277 25761088

[B62] Schroyen G. Vissers J. Smeets A. Gillebert C. R. Lemiere J. Sunaert S. (2021). Blood and neuroimaging biomarkers of cognitive sequelae in breast cancer patients throughout chemotherapy: a systematic review. *Transl. Oncol.* 16:101297. 10.1016/j.tranon.2021.101297 34896851 PMC8681023

[B63] Scott A. R. Stoltzfus K. C. Tchelebi L. T. Trifiletti D. M. Lehrer E. J. Rao P. (2020). Trends in cancer incidence in US adolescents and young adults, 1973-2015. *JAMA Netw. Open* 3:e2027738. 10.1001/jamanetworkopen.2020.27738 33258907 PMC7709088

[B64] SEER (2022). *Breast People Alive with Cancer (U.S. Prevalence) on January 1, 2018. By Age at Prevalence, Female [Online].* SEER. Available online at: https://seer.cancer.gov/ (accessed January 31, 2022).

[B65] Smith A. W. Bellizzi K. M. Keegan T. H. Zebrack B. Chen V. W. Neale A. V. (2013). Health-related quality of life of adolescent and young adult patients with cancer in the United States: the adolescent and young adult health outcomes and patient experience study. *J. Clin. Oncol.* 31 2136–2145. 10.1200/jco.2012.47.3173 23650427 PMC3731979

[B66] Sousa H. Almeida S. Bessa J. Pereira M. G. (2020). The developmental trajectory of cancer-related cognitive impairment in breast cancer patients: a systematic review of longitudinal neuroimaging studies. *Neuropsychol. Rev.* 30 287–309. 10.1007/s11065-020-09441-9 32607817

[B67] Stafford L. Judd F. Gibson P. Komiti A. Mann G. B. Quinn M. (2015). Anxiety and depression symptoms in the 2 years following diagnosis of breast or gynaecologic cancer: prevalence, course and determinants of outcome. *Support Care Cancer* 23 2215–2224. 10.1007/s00520-014-2571-y 25559036

[B68] Suvarna B. Suvarna A. Phillips R. Juster R. P. McDermott B. Sarnyai Z. (2020). Health risk behaviours and allostatic load: a systematic review. *Neurosci. Biobehav. Rev.* 108 694–711. 10.1016/j.neubiorev.2019.12.020 31846655

[B69] Usta Y. Y. (2012). Importance of social support in cancer patients. *Asian Pac. J. Cancer Prev.* 13 3569–3572. 10.7314/apjcp.2012.13.8.3569 23098436

[B70] van der Plas E. Spencer Noakes T. L. Butcher D. T. Weksberg R. Galin-Corini L. Wanstall E. A. (2020). Cognitive and behavioral risk factors for low quality of life in survivors of childhood acute lymphoblastic leukemia. *Pediatr. Res*. 90 419–426. 10.1038/s41390-020-01230-7 33203967 PMC9014848

[B71] Van Esch L. Roukema J. A. Ernst M. F. Nieuwenhuijzen G. A. De Vries J. (2012). Combined anxiety and depressive symptoms before diagnosis of breast cancer. *J Affect Disord* 136 895–901. 10.1016/j.jad.2011.09.012 21975139

[B72] Wagner L. I. Gray R. J. Sparano J. A. Whelan T. J. Garcia S. F. Yanez B. (2020). Patient-reported cognitive impairment among women with early breast cancer randomly assigned to endocrine therapy alone versus chemoendocrine therapy: results from TAILORx. *J. Clin. Oncol.* 38 1875–1886. 10.1200/jco.19.01866 32271671 PMC7280048

[B73] Yi J. C. Syrjala K. L. (2017). Anxiety and depression in cancer survivors. *Med. Clin. North Am.* 101 1099–1113. 10.1016/j.mcna.2017.06.005 28992857 PMC5915316

