# Supplementary material for: Characterizing Early Changes in Quality of Life in Young Women With Breast Cancer
Source: Front Psychol. 2022 May 6;13:871194. doi: 10.3389/fpsyg.2022.871194 (PMC9132041; doi:10.3389/fpsyg.2022.871194)
Supplement: Supplementary file 1 [file Table_1.DOCX]

Supplementary Material

# Table 1: Estimated means and rate of change for overall FACT-B scores across predictors

| **Estimated Group Means and Rate of Change: FACT-B** | | | | | |
| --- | --- | --- | --- | --- | --- |
| **Effect** | **Level** | **Enrollment** | **Change**  **(12-month – Enrollment)** | **Beta**  **(Interaction)** | **P-value** |
| Baseline Clinical Stage (Grouped) | 0-I | 113.1 (104.5, 121.8) | 8.9 (-0.3, 18.2) | Ref | 0.59 |
|  | II-III | 106.7 (100.7, 112.7) | 6.0 (0.2, 11.7) | -3.0 |  |
| Laterality | Left | 112.9 (106.4, 119.4) | 6.5 (-0.3, 13.2) | Ref | 0.97 |
|  | Right | 103.0 (96.2, 109.9) | 6.6 (0.4, 12.8) | 0.2 |  |
| ER (Combined) | Negative | 106.2 (99.2, 113.1) | 4.3 (-2.3, 10.9) | Ref | 0.34 |
|  | Positive | 110.6 (104.1, 117.1) | 8.7 (2.5, 14.8) | 4.4 |  |
| PR (Combined) | Negative | 106.9 (100.1, 113.6) | 4.6 (-1.9, 11.0) | Ref | 0.32 |
|  | Positive | 109.6 (102.7, 116.5) | 9.2 (2.7, 15.6) | 4.6 |  |
| Her2 Status | Neg/Neu | 109.2 (103.0, 115.4) | 5.4 (-0.9, 11.6) | Ref | 0.59 |
|  | Positive | 103.6 (94.9, 112.4) | 7.9 (0.6, 15.2) | 2.6 |  |
| Genetic Susceptibility | No | 107.1 (101.9, 112.2) | 8.3 (3.9, 12.6) | Ref | 0.35 |
|  | Yes | 113.2 (101.2, 125.1) | 3.4 (-7.3, 14.1) | 6.1 |  |
| Fertility Counseling | No | 107.9 (102.2, 113.5) | 7.0 (1.8, 12.1) | Ref | 0.75 |
|  | Yes | 110.3 (100.7, 119.9) | 5.3 (-4.4, 14.9) | -1.7 |  |
| Anxiety or Depression at Dx | No | 111.6 (106.1, 117.2) | 4.9 (0.2, 9.7) | Ref | **0.05** |
|  | Yes | 99.4 (90.7, 108.0) | 12.9 (6.4, 19.5) | 8.0 |  |
| Marital Status (Combined) | Married | 110.9 (104.7, 117.1) | 5.0 (-1.1, 11.1) | -6.1 | 0.22 |
|  | Not Married | 102.5 (93.3, 111.8) | 11.0 (3.4, 18.7) | Ref |  |
| Biological parent history of breast cancer | No | 106.6 (101.0, 112.3) | 8.1 (3.1, 13.1) | Ref | 0.17 |
|  | Yes | 118.8 (107.1, 130.5) | -0.6 (-12.1, 10.8) | -8.7 |  |
| Family History of Cancer (Combined) | No | 108.7 (101.3, 116.2) | 6.2 (-0.4, 12.8) | Ref | 0.87 |
|  | Yes | 108.3 (101.9, 114.7) | 7.0 (0.7, 13.3) | 0.8 |  |
| Most Aggressive Surgery | Lumpectomy | 114.1 (103.1, 125.2) | 1.2 (-9.7, 12.1) | Ref | 0.28 |
|  | Mastectomy | 107.3 (101.9, 112.7) | 7.7 (2.7, 12.6) | 6.5 |  |
| Reconstruction | No | 103.9 (96.3, 111.6) | 5.1 (-2.5, 12.6) | Ref | 0.60 |
|  | Yes | 111.6 (105.6, 117.6) | 7.5 (1.9, 13.2) | 2.5 |  |
| Chemo | No | 116.4 (104.2, 128.7) | 6.7 (-5.4, 18.9) | Ref | 0.96 |
|  | Yes | 106.9 (101.7, 112.0) | 6.4 (1.5, 11.3) | -0.3 |  |
| Targeted Therapy | No | 112.7 (107.0, 118.4) | 5.6 (-0.3, 11.5) | Ref | 0.65 |
|  | Yes | 98.9 (90.7, 107.1) | 7.8 (0.6, 15.0) | 2.1 |  |
| Radiation | No | 109.2 (102.3, 116.0) | 8.3 (1.7, 14.9) | Ref | 0.50 |
|  | Yes | 107.8 (101.1, 114.6) | 5.2 (-1.0, 11.5) | -3.1 |  |
| Hormone Therapy | No | 109.7 (103.5, 116.0) | 3.6 (-2.2, 9.4) | Ref | 0.11 |
|  | Yes | 106.9 (99.3, 114.4) | 10.9 (4.0, 17.8) | 7.3 |  |
| Ovarian Suppression | No | 108.3 (103.0, 113.7) | 6.1 (0.9, 11.3) | Ref | 0.67 |
|  | Yes | 110.0 (98.0, 122.1) | 8.4 (-1.0, 17.8) | 2.3 |  |
| BMI | Mean (26.6) | 108.1 (103.4, 112.9) | 7.4 (3.5, 11.4) | -0.7 | 0.07 |
| Age at Dx | Mean (34.3) | 108.8 (103.9, 113.7) | 6.1 (1.4, 10.7) | -0.4 | 0.35 |

# Table 2 Estimated means and rate of change for physical well-being across predictors

| **Estimated Group Means and Rate of Change: PWB** | | | | | |
| --- | --- | --- | --- | --- | --- |
| **Effect** | **Level** | **Enrollment** | **Change**  **(12-month – Enrollment)** | **Beta**  **(Interaction)** | **P-value** |
| Baseline Clinical Stage (Grouped) | 0-I | 23.9 (21.7, 26.1) | 1.3 (-1.4, 4.0) | Ref | 0.80 |
|  | II-III | 22.0 (20.4, 23.5) | 1.7 (0.0, 3.5) | 0.4 |  |
| Laterality | Left | 23.2 (21.5, 24.8) | 1.5 (-0.5, 3.5) | Ref | 1.00 |
|  | Right | 21.9 (20.2, 23.6) | 1.5 (-0.4, 3.3) | 0.0 |  |
| ER (Combined) | Negative | 22.5 (20.7, 24.3) | 1.2 (-0.8, 3.2) | Ref | 0.68 |
|  | Positive | 22.7 (21.1, 24.3) | 1.7 (-0.1, 3.5) | 0.6 |  |
| PR (Combined) | Negative | 22.7 (21.0, 24.4) | 1.3 (-0.7, 3.2) | Ref | 0.68 |
|  | Positive | 22.4 (20.7, 24.0) | 1.8 (0.0, 3.7) | 0.6 |  |
| Her2 Status | Neg/Neu | 22.8 (21.2, 24.3) | 0.8 (-1.0, 2.7) | Ref | 0.21 |
|  | Positive | 21.4 (19.1, 23.6) | 2.8 (0.3, 5.2) | 1.9 |  |
| Genetic Susceptibility | No | 22.3 (21.0, 23.6) | 1.9 (0.5, 3.3) | Ref | 0.46 |
|  | Yes | 23.5 (20.5, 26.5) | 0.7 (-2.7, 4.0) | 1.2 |  |
| Fertility Counseling | No | 22.0 (20.6, 23.4) | 1.9 (0.4, 3.4) | Ref | 0.24 |
|  | Yes | 24.4 (22.0, 26.7) | 0.0 (-2.8, 2.8) | -1.9 |  |
| Anxiety or Depression at Dx | No | 22.6 (21.2, 24.0) | 1.9 (0.4, 3.5) | Ref | 0.62 |
|  | Yes | 22.1 (19.8, 24.4) | 1.2 (-1.2, 3.6) | -0.7 |  |
| Marital Status (Combined) | Married | 22.6 (21.1, 24.1) | 1.4 (-0.3, 3.1) | -0.5 | 0.74 |
|  | Not Married | 22.4 (20.2, 24.7) | 1.9 (-0.5, 4.3) | Ref |  |
| Biological parent history of breast cancer | No | 22.2 (21.0, 23.5) | 1.9 (0.3, 3.4) | Ref | 0.27 |
|  | Yes | 25.5 (23.0, 28.0) | -0.1 (-3.4, 3.1) | -2.0 |  |
| Family History of Cancer (Combined) | No | 20.9 (19.1, 22.7) | 2.6 (0.6, 4.5) | Ref | 0.15 |
|  | Yes | 23.8 (22.3, 25.4) | 0.6 (-1.2, 2.4) | -2.0 |  |
| Most Aggressive Surgery | Lumpectomy | 22.7 (19.9, 25.4) | 1.0 (-2.1, 4.2) | Ref | 0.74 |
|  | Mastectomy | 22.6 (21.3, 23.9) | 1.6 (0.1, 3.1) | 0.6 |  |
| Reconstruction | No | 20.5 (18.7, 22.2) | 2.2 (0.1, 4.4) | Ref | 0.35 |
|  | Yes | 24.1 (22.7, 25.6) | 0.9 (-0.8, 2.7) | -1.3 |  |
| Chemo | No | 25.5 (22.5, 28.4) | 0.0 (-3.4, 3.5) | Ref | 0.38 |
|  | Yes | 22.1 (20.8, 23.3) | 1.7 (0.2, 3.1) | 1.6 |  |
| Targeted Therapy | No | 23.3 (21.9, 24.8) | 0.9 (-0.8, 2.5) | Ref | 0.22 |
|  | Yes | 21.0 (18.9, 23.1) | 2.6 (0.3, 4.9) | 1.8 |  |
| Radiation | No | 23.0 (21.3, 24.7) | 1.8 (-0.2, 3.8) | Ref | 0.74 |
|  | Yes | 22.2 (20.5, 23.8) | 1.3 (-0.5, 3.1) | -0.5 |  |
| Hormone Therapy | No | 23.2 (21.6, 24.8) | 0.8 (-1.0, 2.6) | Ref | 0.28 |
|  | Yes | 21.8 (20.0, 23.6) | 2.3 (0.3, 4.3) | 1.5 |  |
| Ovarian Suppression | No | 22.6 (21.3, 23.9) | 1.6 (0.0, 3.1) | Ref | 0.82 |
|  | Yes | 22.5 (19.7, 25.4) | 1.2 (-1.5, 4.0) | -0.4 |  |
| BMI | Mean (26.6) | 22.5 (21.3, 23.7) | 1.7 (0.4, 2.9) | -0.1 | 0.23 |
| Age at Dx | Mean (34.3) | 22.6 (21.4, 23.8) | 1.4 (0.0, 2.8) | -0.1 | 0.46 |

# Table 3 Estimated means and rate of change for social well-being across predictors

| **Estimated Group Means and Rate of Change: SWB** | | | | | |
| --- | --- | --- | --- | --- | --- |
| **Effect** | **Level** | **Enrollment** | **Change**  **(12-month – Enrollment)** | **Beta**  **(Interaction)** | **P-value** |
| Baseline Clinical Stage (Grouped) | 0-I | 23.1 (21.2, 25.0) | 1.0 (-1.1, 3.1) | Ref | **0.04** |
|  | II-III | 24.0 (22.7, 25.4) | -1.7 (-3.0, -0.3) | -2.6 |  |
| Laterality | Left | 24.4 (23.0, 25.9) | -0.4 (-2.1, 1.2) | Ref | 0.55 |
|  | Right | 22.3 (20.8, 23.8) | -1.1 (-2.6, 0.4) | -0.7 |  |
| ER (Combined) | Negative | 23.3 (21.7, 25.0) | -0.7 (-2.4, 1.0) | Ref | 0.82 |
|  | Positive | 23.7 (22.2, 25.1) | -0.9 (-2.4, 0.5) | -0.3 |  |
| PR (Combined) | Negative | 23.8 (22.2, 25.4) | -1.1 (-2.8, 0.5) | Ref | 0.66 |
|  | Positive | 23.1 (21.6, 24.6) | -0.6 (-2.1, 0.9) | 0.5 |  |
| Her2 Status | Neg/Neu | 24.1 (22.7, 25.5) | -1.4 (-2.9, 0.1) | Ref | 0.31 |
|  | Positive | 21.9 (19.9, 24.0) | -0.1 (-2.0, 1.8) | 1.2 |  |
| Genetic Susceptibility | No | 23.4 (22.2, 24.6) | -0.7 (-1.8, 0.5) | Ref | 0.78 |
|  | Yes | 23.8 (21.0, 26.6) | -1.0 (-3.8, 1.8) | 0.4 |  |
| Fertility Counseling | No | 23.7 (22.4, 25.0) | -0.9 (-2.1, 0.3) | Ref | 0.81 |
|  | Yes | 23.0 (20.8, 25.2) | -0.6 (-2.9, 1.8) | 0.3 |  |
| Anxiety or Depression at Dx | No | 23.5 (22.2, 24.8) | -1.3 (-2.5, 0.0) | Ref | 0.14 |
|  | Yes | 23.3 (21.2, 25.4) | 0.5 (-1.4, 2.4) | 1.7 |  |
| Marital Status (Combined) | Married | 24.1 (22.7, 25.4) | -0.6 (-2.0, 0.8) | 0.0 | 1.00 |
|  | Not Married | 21.7 (19.7, 23.8) | -0.6 (-2.6, 1.3) | Ref |  |
| Biological parent history of breast cancer | No | 23.0 (21.7, 24.3) | -0.6 (-1.7, 0.6) | Ref | 0.89 |
|  | Yes | 23.8 (21.3, 26.4) | -0.4 (-3.0, 2.2) | 0.2 |  |
| Family History of Cancer (Combined) | No | 24.0 (22.3, 25.7) | -0.8 (-2.4, 0.7) | Ref | 1.00 |
|  | Yes | 23.2 (21.7, 24.6) | -0.8 (-2.4, 0.7) | 0.0 |  |
| Most Aggressive Surgery | Lumpectomy | 24.7 (22.2, 27.2) | -1.9 (-4.5, 0.8) | Ref | 0.40 |
|  | Mastectomy | 23.2 (22.0, 24.5) | -0.6 (-1.8, 0.6) | 1.2 |  |
| Reconstruction | No | 24.1 (22.4, 25.8) | -2.5 (-4.1, -0.9) | Ref | **0.01** |
|  | Yes | 23.2 (21.8, 24.6) | 0.2 (-1.1, 1.5) | 2.7 |  |
| Chemo | No | 22.5 (19.7, 25.3) | 0.8 (-2.1, 3.7) | Ref | 0.23 |
|  | Yes | 23.7 (22.5, 24.9) | -1.1 (-2.3, 0.1) | -1.9 |  |
| Targeted Therapy | No | 24.5 (23.3, 25.8) | -1.3 (-2.6, 0.1) | Ref | 0.31 |
|  | Yes | 21.1 (19.2, 23.0) | -0.1 (-1.9, 1.7) | 1.2 |  |
| Radiation | No | 23.2 (21.7, 24.8) | -0.1 (-1.8, 1.5) | Ref | 0.25 |
|  | Yes | 23.7 (22.2, 25.3) | -1.4 (-2.9, 0.1) | -1.3 |  |
| Hormone Therapy | No | 23.7 (22.3, 25.1) | -0.7 (-2.2, 0.8) | Ref | 0.80 |
|  | Yes | 23.2 (21.5, 24.9) | -1.0 (-2.6, 0.7) | -0.3 |  |
| Ovarian Suppression | No | 23.5 (22.3, 24.7) | -0.8 (-2.0, 0.5) | Ref | 0.85 |
|  | Yes | 23.4 (20.8, 26.0) | -1.0 (-3.2, 1.2) | -0.3 |  |
| BMI | Mean (26.6) | 23.4 (22.3, 24.5) | -0.7 (-1.8, 0.4) | 0.0 | 0.87 |
| Age at Dx | Mean (34.3) | 23.6 (22.5, 24.6) | -0.9 (-2.0, 0.2) | -0.1 | 0.35 |

# Table 4 Estimated means and rate of change for emotional well-being across predictors

| **Estimated Group Means and Rate of Change: EWB** | | | | | |
| --- | --- | --- | --- | --- | --- |
| **Effect** | **Level** | **Enrollment** | **Change**  **(12-month – Enrollment)** | **Beta**  **(Interaction)** | **P-value** |
| Baseline Clinical Stage (Grouped) | 0-I | 16.6 (14.6, 18.6) | 3.2 (1.2, 5.2) | Ref | 0.15 |
|  | II-III | 15.5 (14.1, 16.9) | 1.5 (0.3, 2.8) | -1.7 |  |
| Laterality | Left | 16.1 (14.5, 17.6) | 2.2 (0.7, 3.7) | Ref | 0.55 |
|  | Right | 15.8 (14.2, 17.4) | 1.6 (0.3, 2.9) | -0.6 |  |
| ER (Combined) | Negative | 14.3 (12.7, 15.9) | 2.2 (0.7, 3.7) | Ref | 0.63 |
|  | Positive | 17.4 (15.9, 18.8) | 1.7 (0.4, 3.0) | -0.5 |  |
| PR (Combined) | Negative | 14.1 (12.5, 15.6) | 2.6 (1.1, 4.0) | Ref | 0.23 |
|  | Positive | 17.8 (16.3, 19.3) | 1.3 (0.0, 2.7) | -1.2 |  |
| Her2 Status | Neg/Neu | 15.9 (14.4, 17.3) | 2.0 (0.7, 3.4) | Ref | 0.65 |
|  | Positive | 15.5 (13.4, 17.5) | 1.5 (-0.1, 3.2) | -0.5 |  |
| Genetic Susceptibility | No | 15.6 (14.4, 16.8) | 2.0 (0.9, 3.1) | Ref | 0.19 |
|  | Yes | 17.6 (14.8, 20.4) | 1.4 (-1.3, 4.0) | 2.0 |  |
| Fertility Counseling | No | 16.1 (14.8, 17.4) | 1.6 (0.5, 2.7) | Ref | 0.32 |
|  | Yes | 15.7 (13.4, 17.9) | 2.8 (0.7, 5.0) | 1.2 |  |
| Anxiety or Depression at Dx | No | 16.9 (15.6, 18.1) | 1.7 (0.5, 2.9) | Ref | 0.37 |
|  | Yes | 13.6 (11.5, 15.7) | 2.6 (0.9, 4.4) | 1.0 |  |
| Marital Status (Combined) | Married | 16.1 (14.7, 17.5) | 1.7 (0.4, 3.0) | -0.2 | 0.83 |
|  | Not Married | 16.0 (13.8, 18.1) | 2.0 (0.2, 3.7) | Ref |  |
| Biological parent history of breast cancer | No | 15.8 (14.4, 17.1) | 2.1 (1.1, 3.1) | Ref | 0.60 |
|  | Yes | 16.8 (14.1, 19.4) | 2.8 (0.5, 5.1) | 0.7 |  |
| Family History of Cancer (Combined) | No | 17.4 (15.7, 19.0) | 0.2 (-1.1, 1.4) | Ref | **<.01** |
|  | Yes | 14.8 (13.4, 16.3) | 3.5 (2.2, 4.7) | 3.3 |  |
| Most Aggressive Surgery | Lumpectomy | 17.0 (14.5, 19.5) | 0.3 (-2.1, 2.7) | Ref | 0.17 |
|  | Mastectomy | 15.8 (14.6, 17.1) | 2.2 (1.1, 3.2) | 1.8 |  |
| Reconstruction | No | 15.0 (13.3, 16.7) | 0.9 (-0.6, 2.4) | Ref | 0.10 |
|  | Yes | 16.7 (15.3, 18.1) | 2.5 (1.3, 3.7) | 1.6 |  |
| Chemo | No | 18.5 (15.8, 21.2) | 2.0 (-0.7, 4.6) | Ref | 0.93 |
|  | Yes | 15.5 (14.3, 16.6) | 1.8 (0.8, 2.9) | -0.1 |  |
| Targeted Therapy | No | 16.5 (15.1, 17.8) | 2.0 (0.7, 3.3) | Ref | 0.75 |
|  | Yes | 14.7 (12.7, 16.7) | 1.7 (0.0, 3.3) | -0.3 |  |
| Radiation | No | 15.9 (14.3, 17.5) | 2.5 (1.0, 4.0) | Ref | 0.27 |
|  | Yes | 16.1 (14.5, 17.6) | 1.4 (0.1, 2.7) | -1.1 |  |
| Hormone Therapy | No | 15.2 (13.8, 16.7) | 1.9 (0.5, 3.2) | Ref | 0.96 |
|  | Yes | 17.1 (15.4, 18.8) | 1.9 (0.4, 3.4) | 0.1 |  |
| Ovarian Suppression | No | 15.7 (14.4, 16.9) | 2.2 (1.0, 3.3) | Ref | 0.31 |
|  | Yes | 17.4 (14.7, 20.1) | 1.0 (-0.8, 2.9) | -1.1 |  |
| BMI | Mean (26.6) | 16.0 (14.9, 17.1) | 1.9 (0.9, 2.9) | -0.1 | 0.31 |
| Age at Dx | Mean (34.3) | 16.0 (14.9, 17.1) | 1.8 (0.8, 2.9) | 0.0 | 0.86 |

# Table 5 Estimated means and rate of change for functional well-being across predictors

| **Estimated Group Means and Rate of Change: FWB** | | | | | |
| --- | --- | --- | --- | --- | --- |
| **Effect** | **Level** | **Enrollment** | **Change**  **(12-month – Enrollment)** | **Beta**  **(Interaction)** | **P-value** |
| Baseline Clinical Stage (Grouped) | 0-I | 20.9 (18.7, 23.2) | 3.3 (1.2, 5.4) | Ref | 0.71 |
|  | II-III | 19.3 (17.7, 20.9) | 2.8 (1.5, 4.1) | -0.5 |  |
| Laterality | Left | 21.0 (19.3, 22.8) | 2.8 (1.2, 4.5) | Ref | 0.76 |
|  | Right | 17.9 (16.0, 19.7) | 3.2 (1.8, 4.6) | 0.3 |  |
| ER (Combined) | Negative | 19.0 (17.1, 20.9) | 3.1 (1.4, 4.7) | Ref | 0.95 |
|  | Positive | 20.1 (18.4, 21.8) | 3.0 (1.6, 4.4) | -0.1 |  |
| PR (Combined) | Negative | 19.3 (17.4, 21.1) | 3.2 (1.6, 4.8) | Ref | 0.80 |
|  | Positive | 19.7 (17.9, 21.5) | 3.0 (1.5, 4.4) | -0.3 |  |
| Her2 Status | Neg/Neu | 19.6 (17.9, 21.3) | 2.7 (1.3, 4.2) | Ref | 0.39 |
|  | Positive | 18.5 (16.1, 20.9) | 3.7 (2.0, 5.5) | 1.0 |  |
| Genetic Susceptibility | No | 19.2 (17.8, 20.6) | 3.5 (2.4, 4.6) | Ref | 0.20 |
|  | Yes | 21.4 (18.3, 24.6) | 1.1 (-1.6, 3.7) | 2.2 |  |
| Fertility Counseling | No | 19.5 (18.0, 21.0) | 3.0 (1.8, 4.2) | Ref | 0.80 |
|  | Yes | 19.9 (17.4, 22.5) | 3.3 (1.0, 5.6) | 0.3 |  |
| Anxiety or Depression at Dx | No | 20.3 (18.8, 21.8) | 2.3 (1.1, 3.4) | Ref | **<.01** |
|  | Yes | 17.6 (15.2, 20.0) | 5.2 (3.5, 6.9) | 2.9 |  |
| Marital Status (Combined) | Married | 20.9 (19.3, 22.5) | 2.3 (0.9, 3.7) | -2.1 | 0.07 |
|  | Not Married | 16.8 (14.4, 19.3) | 4.4 (2.6, 6.2) | Ref |  |
| Biological parent history of breast cancer | No | 18.9 (17.3, 20.4) | 3.5 (2.3, 4.7) | Ref | 0.10 |
|  | Yes | 22.4 (19.2, 25.6) | 1.1 (-1.7, 3.8) | -2.5 |  |
| Family History of Cancer (Combined) | No | 19.2 (17.3, 21.2) | 3.3 (1.8, 4.8) | Ref | 0.61 |
|  | Yes | 19.9 (18.2, 21.6) | 2.8 (1.2, 4.3) | -0.6 |  |
| Most Aggressive Surgery | Lumpectomy | 20.0 (17.1, 22.8) | 3.2 (0.6, 5.8) | Ref | 0.89 |
|  | Mastectomy | 19.5 (18.0, 20.9) | 3.0 (1.8, 4.2) | -0.2 |  |
| Reconstruction | No | 17.8 (15.9, 19.8) | 3.9 (2.2, 5.6) | Ref | 0.18 |
|  | Yes | 20.8 (19.2, 22.4) | 2.5 (1.1, 3.8) | -1.5 |  |
| Chemo | No | 22.3 (19.1, 25.5) | 1.2 (-1.6, 4.0) | Ref | 0.17 |
|  | Yes | 19.1 (17.7, 20.5) | 3.3 (2.2, 4.4) | 2.1 |  |
| Targeted Therapy | No | 20.5 (19.0, 22.0) | 2.8 (1.5, 4.2) | Ref | 0.65 |
|  | Yes | 17.3 (15.1, 19.6) | 3.3 (1.6, 5.1) | 0.5 |  |
| Radiation | No | 19.7 (17.8, 21.5) | 3.2 (1.6, 4.8) | Ref | 0.77 |
|  | Yes | 19.5 (17.7, 21.3) | 2.9 (1.5, 4.3) | -0.3 |  |
| Hormone Therapy | No | 19.9 (18.2, 21.6) | 2.8 (1.4, 4.3) | Ref | 0.66 |
|  | Yes | 19.2 (17.2, 21.2) | 3.3 (1.7, 4.9) | 0.5 |  |
| Ovarian Suppression | No | 19.3 (17.9, 20.7) | 3.4 (2.1, 4.6) | Ref | 0.32 |
|  | Yes | 20.9 (17.8, 24.0) | 2.2 (0.2, 4.2) | -1.2 |  |
| BMI | Mean (26.6) | 19.6 (18.3, 20.8) | 3.1 (2.1, 4.2) | -0.1 | 0.57 |
| Age at Dx | Mean (34.3) | 19.6 (18.3, 20.9) | 3.0 (1.9, 4.0) | -0.1 | 0.55 |

# Table 6 Estimated means and rate of change for the breast cancer scale across predictors

| **Estimated Group Means and Rate of Change: BCS** | | | | | |
| --- | --- | --- | --- | --- | --- |
| **Effect** | **Level** | **Enrollment** | **Change**  **(12-month – Enrollment)** | **Beta**  **(Interaction)** | **P-value** |
| Baseline Clinical Stage (Grouped) | 0-I | 29.1 (25.9, 32.3) | -0.3 (-4.2, 3.6) | Ref | 0.90 |
|  | II-III | 26.5 (24.3, 28.7) | 0.0 (-2.6, 2.5) | 0.3 |  |
| Laterality | Left | 28.7 (26.4, 31.0) | -0.1 (-2.9, 2.7) | Ref | 0.91 |
|  | Right | 25.8 (23.3, 28.2) | -0.3 (-3.1, 2.4) | -0.2 |  |
| ER (Combined) | Negative | 27.2 (24.8, 29.7) | -1.6 (-4.4, 1.2) | Ref | 0.15 |
|  | Positive | 27.5 (25.2, 29.8) | 1.2 (-1.4, 3.8) | 2.8 |  |
| PR (Combined) | Negative | 27.2 (24.8, 29.6) | -1.5 (-4.2, 1.2) | Ref | 0.12 |
|  | Positive | 27.4 (24.9, 29.8) | 1.5 (-1.2, 4.2) | 3.0 |  |
| Her2 Status | Neg/Neu | 27.5 (25.2, 29.7) | -0.4 (-3.1, 2.2) | Ref | 0.91 |
|  | Positive | 26.4 (23.3, 29.5) | -0.2 (-3.5, 3.1) | 0.3 |  |
| Genetic Susceptibility | No | 27.0 (25.1, 28.8) | 0.3 (-1.7, 2.3) | Ref | 0.56 |
|  | Yes | 28.3 (24.0, 32.7) | -0.1 (-4.9, 4.6) | 1.4 |  |
| Fertility Counseling | No | 27.4 (25.4, 29.4) | -0.2 (-2.4, 2.1) | Ref | 0.97 |
|  | Yes | 27.2 (23.8, 30.7) | -0.1 (-4.1, 4.0) | 0.1 |  |
| Anxiety or Depression at Dx | No | 28.7 (26.8, 30.6) | -0.8 (-2.9, 1.3) | Ref | 0.07 |
|  | Yes | 23.4 (20.4, 26.4) | 2.6 (-0.4, 5.6) | 3.4 |  |
| Marital Status (Combined) | Married | 28.0 (25.8, 30.2) | -1.5 (-4.1, 1.0) | -4.9 | **0.02** |
|  | Not Married | 25.7 (22.5, 28.9) | 3.3 (0.0, 6.7) | Ref |  |
| Biological parent history of breast cancer | No | 27.1 (25.2, 29.1) | 0.8 (-1.5, 3.0) | Ref | 0.14 |
|  | Yes | 29.5 (25.4, 33.6) | -3.3 (-8.2, 1.6) | -4.1 |  |
| Family History of Cancer (Combined) | No | 28.1 (25.5, 30.7) | -1.2 (-4.1, 1.8) | Ref | 0.36 |
|  | Yes | 26.8 (24.6, 29.1) | 0.7 (-2.0, 3.4) | 1.8 |  |
| Most Aggressive Surgery | Lumpectomy | 29.5 (25.5, 33.5) | -1.2 (-5.8, 3.4) | Ref | 0.65 |
|  | Mastectomy | 26.9 (25.0, 28.8) | 0.0 (-2.2, 2.2) | 1.2 |  |
| Reconstruction | No | 27.1 (24.3, 29.8) | -1.4 (-4.6, 1.8) | Ref | 0.33 |
|  | Yes | 27.6 (25.4, 29.7) | 0.6 (-1.8, 3.0) | 2.0 |  |
| Chemo | No | 28.7 (24.3, 33.1) | 1.8 (-3.3, 6.9) | Ref | 0.39 |
|  | Yes | 27.1 (25.3, 28.9) | -0.6 (-2.7, 1.5) | -2.4 |  |
| Targeted Therapy | No | 28.6 (26.5, 30.6) | -0.4 (-2.9, 2.1) | Ref | 0.83 |
|  | Yes | 24.7 (21.8, 27.6) | 0.0 (-3.3, 3.3) | 0.4 |  |
| Radiation | No | 27.8 (25.4, 30.2) | 0.7 (-2.2, 3.5) | Ref | 0.45 |
|  | Yes | 26.9 (24.5, 29.3) | -0.8 (-3.6, 1.9) | -1.5 |  |
| Hormone Therapy | No | 28.2 (26.0, 30.4) | -1.6 (-4.1, 0.8) | Ref | 0.06 |
|  | Yes | 26.1 (23.5, 28.8) | 2.1 (-0.8, 5.0) | 3.7 |  |
| Ovarian Suppression | No | 27.6 (25.7, 29.5) | -0.4 (-2.6, 1.7) | Ref | 0.53 |
|  | Yes | 26.3 (22.1, 30.5) | 1.1 (-3.2, 5.3) | 1.5 |  |
| BMI | Mean (26.6) | 27.2 (25.5, 28.9) | 0.3 (-1.5, 2.1) | -0.3 | 0.14 |
| Age at Dx | Mean (34.3) | 27.4 (25.7, 29.2) | -0.3 (-2.3, 1.7) | -0.2 | 0.45 |
